# Rumen fermentation and microbial community composition influenced by live *Enterococcus faecium* supplementation

**DOI:** 10.1186/s13568-019-0848-8

**Published:** 2019-07-30

**Authors:** Lovelia L. Mamuad, Seon Ho Kim, Ashraf A. Biswas, Zhongtang Yu, Kwang-Keun Cho, Sang-Bum Kim, Kichoon Lee, Sang Suk Lee

**Affiliations:** 1 Department of Animal Science and Technology, Sunchon National University, Jeonnam, South Korea; 2 Department of Animal Sciences, Ohio State University, Columbus, OH USA; 3 Department of Animal Resources Technology, Gyeongnam National University of Science and Technology, Jinju, Gyeongnam Republic of Korea; 4 Dairy Science Division, National Institute of Animal Science, Rural Development Administration, Cheonan, Chungnam Republic of Korea

**Keywords:** Bar-coded pyrosequencing, *Enterococcus faecium*, In vitro rumen fermentation, Methane concentration, Microbial diversity

## Abstract

Supplementation of appropriate probiotics can improve the health and productivity of ruminants while mitigating environmental methane production. Hence, this study was conducted to determine the effects of *Enterococcus faecium* SROD on in vitro rumen fermentation, methane concentration, and microbial population structure. Ruminal samples were collected from ruminally cannulated Holstein–Friesian cattle, and 40:60 rice straw to concentrate ratio was used as substrate. Fresh culture of *E. faecium* SROD at different inclusion rates (0, 0.1%, 0.5%, and 1.0%) were investigated using in vitro rumen fermentation system. Addition of *E. faecium* SROD had a significant effect on total gas production with the greatest effect observed with 0.1% supplementation; however, there was no significant influence on pH. Supplementation of 0.1% *E. faecium* SROD resulted in the highest propionate (*P *= 0.005) but the lowest methane concentration (*P *= 0.001). In addition, acetate, butyrate, and total VFA concentrations in treatments were comparatively higher than control. Bioinformatics analysis revealed the predominance of the bacterial phyla *Bacteroidetes* and *Firmicutes* and the archaeal phylum *Euryarchaeota*. At the genus level, *Prevotella* (15–17%) and *Methanobrevibacter* (96%) dominated the bacterial and archaeal communities of the in vitro rumen fermenta, respectively. Supplementation of 0.1% *E. faecium* SROD resulted in the highest quantities of total bacteria and *Ruminococcus flavefaciens*, whereas 1.0% *E. faecium* SROD resulted in the highest contents of total fungi and *Fibrobacter succinogenes.* Overall, supplementation of 0.1% *E. faecium* SROD significantly increased the propionate and total volatile fatty acids concentrations but decreased the methane concentration while changing the microbial community abundance and composition.

## Introduction

Probiotics are beneficial live microorganisms that are used as feed supplements for improving the intestinal microbial balance as well as growth performance in livestock. The term “probiotics” has been amended by the Food and Agriculture Organization/World Health Organization to “live microorganisms, which, when administered in adequate amounts, confer a health benefit on the host” (Fuller 1989). Accordingly, probiotics have been used to modulate the balance and activities of the gastrointestinal microbiota and have been developed as functional foods (Uyeno et al. 2015) as well as growth promoters to replace the widely used antibiotic and synthetic chemical-based feed supplements (Fuller 1989).

The rumen microbiome is composed of complex and diverse groups of microorganisms, which are responsible for converting fibrous plant materials into energy used by the ruminants. These microorganisms thus play an important role in animal health and productivity, food safety, and the environment. The rumen microbial community, diversity, and quantity vary depending on the host’s dietary composition and dry matter intake. Hence, supplementation of specific probiotics to ruminants can increase microbial diversity and enhance the proportion of beneficial microbes in the community. Probiotics belong to a wide variety of yeasts, *Bacillus*, and lactic acid bacteria (*Lactobacillus*, *Bifidobacterium*, and *Enterococcus*), which are now commonly used for human as well as animal consumption.

*Enterococcus* is one of the main genera of lactic acid bacteria, which has been used as probiotics for decades. Enterococci are ubiquitous and facultative anaerobes, which means that they can be easily cultivated and proliferate under aerobic conditions during production as well as under the anaerobic conditions found inside the rumen. Moreover, enterococci are resistant to gastric juices and bile salts (Rossi et al. 2003; Li et al. 2019) and produce useful enzymes (Sarantinopoulos et al. 2001), vitamin B12 (Li et al. 2017) and enterocin (an antimicrobial compound) (Yang et al. 2012, 2018), and inhibit harmful microorganisms (Arena et al. 2018; Mansour et al. 2018). *Enterococcus faecium* helps in maintaining the activity of lactate-utilizing bacteria and stimulates the growth of rumen microbes, which can, in turn, increase the glucogenic propionate energy supply for host ruminants (Pang et al. 2014), while also producing antimicrobial agents (Lauková et al. 1993; Wang et al. 2018).

We previously isolated an *E. faecium* SROD strain (KCCM11098P) (Kim et al. 2016) that showed promise as a fumarate reductase-producing enterococci bacterium, and also resulted in enhanced production of total volatile fatty acids (VFAs) while decreasing the concentration of methane during rumen fermentation in vitro. However, the effects of *E. faecium* SROD on the ruminal microbiome remain unclear. Therefore, in the present study, we applied molecular techniques of quantitative real-time polymerase chain reaction (qRT-PCR) and pyrosequencing to determine these effects and gain a better general understanding of rumen microbes’ symbiosis and functions. In particular, we added *E. faecium* SROD at different inclusion rates to an in vitro rumen fermentation system and determined the effects on methane concentration, microbial diversity, and population structure.

## Materials and methods

### Cultivation of *E. faecium* SROD

*Enterococcus faecium* SROD KCCM11098P, which was previously isolated in our laboratory (Kim et al. 2016), was used in this study. A frozen stock culture of *E. faecium* SROD was thawed and re-cultivated at a 1% inoculum using deMan, Rogosa, and Sharpe broth (Becton–Dickinson and Company, Difco, Sparks, MD, USA), and then incubated in a horizontal shaking incubator (120 rpm) (Hanbaek Scientific Co., Korea) at 37 °C. *E. faecium* SROD was then subcultured three times on the same medium to ensure full activity. The cell growth was monitored based on an optical density value at 600 nm of approximately 1.50, which is equivalent to 7.0 × 10^8^ colony-forming units (CFU)/mL.

### Rumen fluid collection and in vitro fermentation

All animal care procedures conducted for this study followed protocols approved by the Sunchon National University Committee on Animal Care. Three ruminally cannulated Holstein–Friesian cows with body weights of 600 ± 47 kg that were fed twice daily with feed concentrate (NongHyup Co., Anseong, Korea) and rice straw (2:8 ratio) were used in this study. Three hours after morning feeding, the ruminal contents were collected, strained through four layers of surgical gauze, placed in amber bottles, maintained at 39 °C, and then immediately transported to the laboratory. Asanuma buffer used in this study was composed of (per L) 0.45 g K_2_HPO_4_, 0.45 g KH_2_PO_4_, 0.9 g (NH_4_)_2_SO_4_, 0.12 g CaCl_2_·2H_2_0, 0.19 g MgSO_4_·7H_2_O, 1.0 g trypticase peptone (BBL; Becton–Dickinson), 1.0 g yeast extract (Difco Laboratories, MI, USA), and 0.6 g cysteine HCl (Asanuma et al. 1999). It was prepared, autoclaved at 121 °C for 15 min, maintained in a 39 °C water bath, flushed with N_2_ for 30 min and continuously flushed until it was transferred to serum bottles. The pH was adjusted to 6.9 using 10 N NaOH. The mixed buffered rumen fluid (1:3 rumen fluid:buffer ratio) was anaerobically transferred (100 mL) to 160 mL serum bottles containing the substrates. The substrates were grinded and sieved to 1 mm particle size and then 1 g dry matter of rice straw and concentrate at 40:60 ratio was put into the serum bottles. The following inocula treatments were conducted under a stream of O_2_-free N_2_: no addition (control; Con), and 0.1% (T1), 0.5% (T2), and 1.0% (T3) supplementation of *E. faecium* SROD (7.0 × 10^8^ colony-forming units (CFU)/mL). The bottles were then sealed and incubated at 39 °C (Mamuad et al. 2014). Five replicates were established for all treatments and incubation times.

### In vitro rumen fermentation parameters

In vitro rumen parameters were sampled in each serum bottle at the end of each incubation period. Total gas was measured using a press and sensor machine (Laurel Electronics, Inc., Costa Mesa, CA, USA), and the pH was determined using a Pinnacle series M530p meter (Schott Instruments, Mainz, Germany). Gas samples were collected for determination of methane concentration, and the in vitro rumen fermenta was collected for ammonia–nitrogen (NH_3_–N), VFA, and molecular analyses. One millilitre of gas sample contained in vacuum tubes was used to determine the methane concentration through gas chromatography (GC; HP 5890 system, Agilent Technologies, Foster City, CA, USA) with a thermal conductivity detector and an HP stainless packed GC Porapak Q 80/100 outer dimension 1/8 in × inner dimension 2.0 mm, length 3.05 m (10 ft) with 200 °C inlet, 200 °C detector, 40 °C oven temperature, and 3 mL/min N_2_ carrier gas flow. Estimation of the amount of methane produced was conducted using the formula described by Ørskov and McDonald (1979). Peaks identification and standards analyses were performed using the procedure described by Kim et al. (2016). Gas standards of known composition were used to identify the peaks. Standards with R^2^ = 0.999 were prepared prior to sample analysis.

Samples were measured from each of the serum bottles at different incubation times. Two 1-mL in vitro rumen fermenta from each serum bottles were immediately centrifuged after sampling at 16,609×*g* for 10 min at 4 °C using a Micro 17TR centrifuge (Hanil Science Industrial Co. Ltd., Incheon, Korea). Then, supernatant and pellet were separated, kept in 1.5 Eppendorf tubes and stored at − 80 °C until subjected to NH_3_–N, VFA, and molecular analyses. The supernatant was used for determination of NH_3_–N (Chaney and Marbach 1962) and VFA concentrations (Kim et al. 2016) with spectrophotometry (Libra S22 spectrophotometer, Biochrom Ltd., Cambridge, UK) and high-performance liquid chromatography (HPLC; Agilent Technologies 1200 series, USA), respectively. The samples for VFA analysis were filtered through 0.2-μm Millipore filters. HPLC had a UV detector set at 210 and 220 nm while MetaCarb 87H (300 × 7.8 mm; Agilent, Germany) column was used in the determination of fermentation products with the application of 0.0085 N H_2_SO_4_ solvent as a buffer at a flow rate of 0.6 mL/min and a column temperature of 35 °C. This was done according to the methods of Tabaru et al. (1988) and Han et al. (2005). Standard was made at 0.999 (R^2^) before analysis. Standards with R^2^ = 0.999 were prepared prior to sample analysis. The VFA concentration in mM was calculated in ppm divided by the molecular weight.

### qRT-PCR

Total genomic deoxyribonucleic acid (DNA) from the rumen pellets was extracted using a Fast-DNA spin kit (MPbio) according to the manufacturer instructions. The general bacteria, general fungi, methanogens, protozoa, *Fibrobacter succinogenes*, and *Ruminococcus flavefaciens* were enumerated using qRT-PCR on the DNA extracted from the in vitro rumen fermenta using the primers reported in Denman and McSweeney (2006) and Denman et al. (2007) (Table 1). Amplification was performed in triplicates using the Eco Real-Time PCR System (Illumina, USA) with QuantiSpeed SYBR no-Rox Kit (PhileKorea, Korea) in final reaction volumes of 20 μL.

**Table 1:** Real time PCR primers used for the quantification of microbial population

Target gene	Primer sequence	Length	Initial denaturation	Denaturation	Annealing	Extension	Cycles	Reference
General bacteria								Denman and McSweeney (2006)
F sequence (5′-3′)	CGGCAACGAGCGCAACCC	130	95 °C 2 min	95 °C 15 s	60 °C 60 s	72 °C 30 s	40	
R sequence (5′-3′)	CCATTGTAGCACGTGTGTAGCC							
General anaerobic fungi								
F sequence (5′-3′)	GAGGAAGTAAAAGTCGTAACAAGGTTTC	120	95 °C 2 min	95 °C 15 s	60 °C 60 s	72 °C 30 s	40	
R sequence (5′-3′)	CAAATTCACAAAGGGTAGGATGATT							
Methanogens								
F sequence (5′-3′)	TTCGGTGGATCDCARAGRGC	140	95 °C 2 min	95 °C 15 s	60 °C 60 s	72 °C 30 s	40	
R sequence (5′-3′)	GBARGTCGWAWCCGTAGAATCC							
*Fibrobacter succinogenes*								
F sequence (5′-3′)	GTTCGGAATTACTGGGCGTAAA	121	95 °C 2 min	95 °C 15 s	60 °C 60 s	72 °C 30 s	40	
R sequence (5′-3′)	CGCCTGCCCCTGAACTATC							
*Ruminococcus flavefaciens*								
F sequence (5′-3′)	CGAACGGAGATAATTTGAGTTTACTTAGG	132	95 °C 2 min	95 °C 15 s	60 °C 60 s	72 °C 30 s	40	
R sequence (5′-3′)	CGGTCTCTGTATGTTATGAGGTATTACC							

### Bar-coded pyrosequencing, PCR, and data analysis

The amplification of bacterial and archaeal 16S rRNA genes for bar-coded pyrosequences and subsequent data analysis were performed according to the procedure described by Lee et al. (2012). The primer sets used for amplification were Bac9F (5′-adaptor B-AC-GAG TTT GAT CMT GGC TCA G-3′)/Bac541R (5′-adaptor A-*X*-AC-WTT ACC GCG GCT GG-3′) and Arc21F (5′-adaptor B-GA-TCC GGT TGA TCC YGC CGG-3′)/Arc519R (5′-adaptor A-*X*-GA-GGT DTT ACC GCG GCK GCT G-3′) (Delong 1992; Sørensen and Teske 2006; Roesch et al. 2007; Chun et al. 2010). Unique 7–11 bp barcode sequences, denoted as “X” in the primer sequences above, were inserted between the 454 Life Sciences adaptor A sequence and the common linkers AC and GA. The polymerase chain reaction (PCR) products were purified and quantified using a PCR purification kit (Solgent, Korea) and an enzyme-linked immunosorbent assay reader equipped with a Take3 multivolume plate, respectively. Equal amounts of purified PCR amplicons from each sample were prepared as a composite DNA sample. The samples were sent to Macrogen (Korea) for pyrosequencing using a 454 GS-FLX Titanium system (Roche, Germany), and the sequencing data were analyzed using the RDP pyrosequencing pipeline (http://pyro.cme.msu.edu/) (Cole et al. 2009). The aligned sequences were clustered into operational taxonomic units (OTUs), defined at 97% similarity, using the complete-linkage clustering tool. The Shannon–Weaver index (Shannon and Weaver 1964), Chao 1 biodiversity indices (Chao 1987), and evenness index and rarefaction analyses were determined using the RDP pyrosequencing pipeline. In addition, the processed sequences were taxonomically classified using the RDP naive Bayesian rRNA classifier (Wang et al. 2007) based on an 80% confidence threshold.

### Statistical analyses

Data were statistically evaluated using Proc Glimmix for a complete randomized design. The experiment was done twice and the control and treatments were conducted in five replicates. Least square means was used to identify differences among control and treatments. Orthogonal contrasts were used to examine the differences between the control and treatment groups. The linear and quadratic effects of *E. faecium* SROD supplementation were analyzed using orthogonal polynomial coefficients to describe the functional relationships among the control and treatment levels. *P* ≤ 0.05 indicated statistical significance. All analyses were carried out using Statistical Analysis Systems (SAS) software version 9.4 (SAS Institute 2012).

## Results

### Effects of *E. faecium* SROD supplementation on rumen fermentation in vitro

The volume of total gas produced was found highest (*P* = 0.017) in supplementation of 0.1% *E. faecium* SROD with 59.45 mL and lowest in control with 55.15 mL (Table 2). Although the addition of *E. faecium* SROD at increasing inclusion rates tended to result in a lower pH value than the control, the difference was not statistically significant. NH_3_–N concentrations were also comparable among the control and treatment groups. However, the methane concentration was linearly correlated (*P* = 0.001) with *E. faecium* SROD addition, and was lowest (*P* = 0.001) with supplementation of 0.1%, followed by 0.5% *E. faecium* SROD, and was highest in the control and 1.0% *E. faecium* SROD groups with no significant difference between them. Addition of 0.1% *E. faecium* SROD resulted in the lowest carbon dioxide (*P* = 0.053) with 3.70 mM/mL but the highest (*P* < 0.001, *P* = 0.005) concentrations of total VFAs and propionate with 55.40 mM and 14.15 mM, respectively (Table 3). Acetate concentration increased (*P* < 0.001) with increasing inclusion rate of *E. faecium* SROD with linearly (*P* < 0.020) and quadratically (*P* < 0.043) correlation of the concentration and inclusion rate. Butyrate (*P* < 0.018) and total VFA (*P* < 0.001) concentrations were comparatively higher than control while propionate (*P* < 0.005) concentration was found the highest in addition of 0.1% *E. faecium* SROD.

**Table 2:** Total gas, pH, ammonia–nitrogen, methane, and carbon dioxide concentrations during in vitro rumen fermentation (12 h)

Parameters	Treatments				SEM	*P*-value		
	Con	T1	T2	T3		Treatment	Linear	Quadratic
Total gas (mL)	55.15^b^	59.45^a^	56.36^ab^	58.16^ab^	0.869	0.017	0.535	0.419
pH	5.43	5.39	5.38	5.38	0.083	0.083	0.037	0.734
Ammonia–nitrogen (mM)	22.28	22.63	20.94	23.16	0.790	0.386	0.271	0.663
Methane (mM/mL)	11.20^a^	9.12^b^	9.93^b^	10.27^ab^	0.238	0.001	0.001	0.074
Carbon dioxide (mM/mL)	4.18^ab^	3.70^b^	4.09^ab^	4.30^a^	0.145	0.053	0.035	0.555

*Con* control (no addition), *T1* 0.1% *E. faecium*, *T2* 0.5% *E. faecium*, *T3* 1.0% *E. faecium*, *SEM* standard error of the mean

Different superscript letters indicate a statistically significant difference. *P*-value, calculated probability

**Table 3:** Volatile fatty acid concentrations during in vitro rumen fermentation (12 h)

Parameters	Treatments				SEM	*P*-value		
	Con	T1	T2	T3		Treatment	Linear	Quadratic
Acetate (mM)	32.52^c^	33.91^b^	34.97^ab^	35.66^a^	0.286	< 0.001	0.020	0.043
Propionate (mM)	9.97^b^	14.15^a^	10.83^b^	10.39^b^	0.180	0.005	0.382	0.013
Butyrate (mM)	5.87^b^	7.20^a^	7.41^a^	7.38^a^	0.295	0.018	0.042	0.142
A/P ratio (mM)	3.11	2.98	3.48	3.21	0.211	0.474	0.627	0.680
Total VFA (mM)	49.19^b^	55.40^a^	55.38^a^	54.17^a^	0.583	< 0.001	0.006	0.039

*Con* control (no addition), *T1* 0.1% *E. faecium*, *T2* 0.5% *E. faecium*, *T3* 1.0% *E. faecium*, *SEM* standard error of the mean

Different superscript letters indicate a statistically significant difference. *P*-value, calculated probability

### Effects of *E. faecium* SROD supplementation on the in vitro rumen microbial community composition and abundance

Comparable quantities of general bacteria were observed among the control and treatment groups (Table 4). Between the cellulolytic bacteria determined, there were more log copies of *F. succinogenes* than *R. flavefaciens.* However, supplementation of 0.1% *E. faecium* SROD resulted in the significantly highest quantities of general fungi (*P* = 0.026), *F. succinogenes* (*P* = 0.010), and *R. flavefaciens* (*P* = 0.008). The control group had the lowest quantities of general fungi (*P* = 0.026) and *F. succinogenes* (*P* = 0.010) but the highest log copy numbers of methanogens (*P* = 0.048), which showed a significant linear decrease with increasing supplementation of *E. faecium* SROD. In addition, supplementation of 0.1% *E. faecium* SROD resulted in lower quantities of methanogen (*P* = 0.048) than control.

**Table 4:** Quantification of general bacteria, general fungi, methanogens, *Fibrobacter succinogenes*, and *Ruminococcus flavefaciens* by real-time PCR

Target genes	Treatments (log_10_ copies number)				SEM	*P*-value		
	Con	T1	T2	T3		Treatment	Linear	Quadratic
General bacteria	8.35	8.41	8.34	8.37	7.355	0.678	0.956	0.350
General anaerobic fungi	3.60^c^	4.01^a^	3.82^bc^	3.97^ab^	2.875	0.026	0.101	0.008
Methanogens	1.44^a^	1.19^b^	1.08^b^	1.01^b^	0.454	0.048	0.032	0.085
*F. succinogenes*	4.37^c^	5.21^a^	4.83^bc^	4.93^b^	3.958	0.010	0.028	0.005
*R. flavefaciens*	2.29^b^	3.18^a^	2.83^b^	2.72^b^	2.060	0.008	0.017	0.012

*Con* control (no addition), *T1* 0.1% *E. faecium*, *T2* 0.5% *E. faecium*, *T3* 1.0% *E. faecium*, *SEM* standard error of the mean

Different superscript letters indicate a statistically significant difference. *P*-value, calculated probability

The barcoded pyrosequencing results of 24 PCR amplicons (NCBI SRA accession PRJNA505970; NCBI Temporary Submission ID: SUB4770572) of the 16S rRNA genes for the bacterial and archaeal communities are shown in Tables 5 and 6, respectively. After filtering, quality control, and chimera removal, the average number of reads, number of operational taxonomic units (OTUs), Chao index, Shannon–Weaver index, evenness, and average read length were 4238.13, 2213.25, 6573.85, 7.15, 0.93, and 476.33 for bacterial communities, and were 4177.50, 116.92, 144.80, 2.93, 0.62, and 490.67 for archaeal communities, respectively (Tables 5 and 6). Rarefaction lines in all samples extended all the way to the right end of the axis.

**Table 5:** Summary of the pyrosequencing data and statistical analysis of bacterial communities of *Enterococcus faecium* SROD

Treatments	No. of reads	No. of OTUs	Chao	Shannon–Weaver index (H’)	Evenness	Avg. read length
Control	4340.00	2228.50	6333.04	7.16	0.93	475.33
0.10%	4137.00	2175.50	6498.96	7.13	0.93	478.00
0.50%	3959.50	2056.50	6420.18	7.06	0.93	476.67
1.00%	4516.00	2392.50	7043.22	7.24	0.93	475.33

*OTU* operational taxonomic units

OTUs were calculated by the RDP pipeline with a 97% OTU cut-off of the 16S rRNA gene sequences. Diversity indices of the microbial communities and numbers of phyla and genera were calculated using the RDP pyrosequencing pipeline based on the 16S rRNA gene sequences

**Table 6:** Summary of the pyrosequencing data and statistical analysis of archaeal communities of *Enterococcus faecium* SROD

Treatments	No. of reads	No. of OTUs	Chao	Shannon–Weaver index (H’)	Evenness	Avg read length
Control	2723.33	102.00	125.95	2.92	0.63	490.67
0.10%	5105.67	126.67	145.14	2.95	0.61	487.67
0.50%	4866.33	122.67	164.85	2.94	0.61	493.33
1.00%	4014.67	116.33	143.26	2.91	0.61	491.00

*OTU* operational taxonomic units

OTUs were calculated by the RDP pipeline with a 97% OTU cut-off of the 16S rRNA gene sequences. Diversity indices of the microbial communities and numbers of phyla and genera were calculated using the RDP pyrosequencing pipeline based on the 16S rRNA gene sequences

**Fig. 1:**
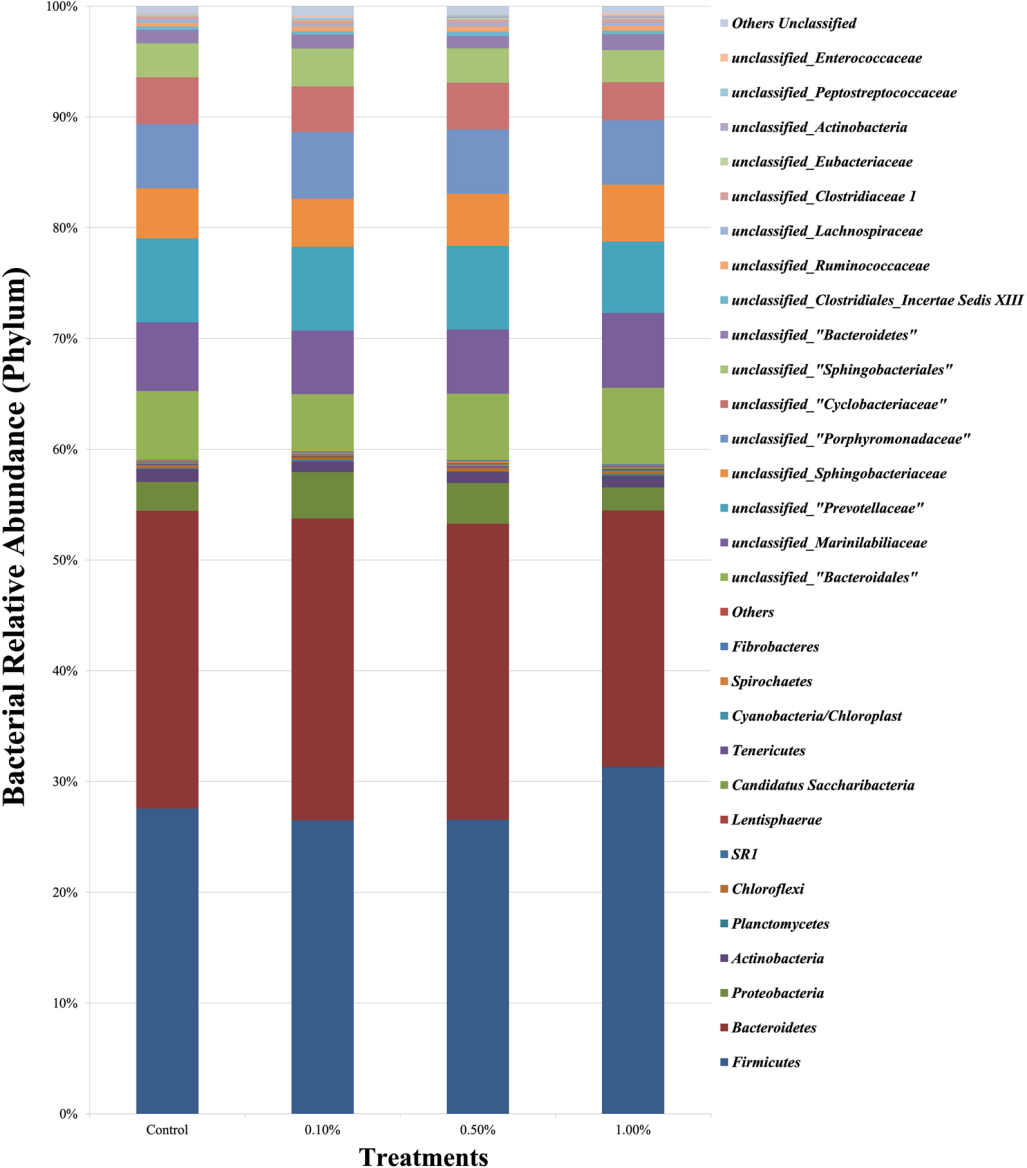
Bacterial phylum-level compositions of the control and *Enterococcus faecium* SROD-supplemented rumen fermenta. The data portray phylum-level 16S rRNA pyrotagged gene sequences. Sequences were classified using the RDP naive Bayesian rRNA Classifier with an 80% confidence threshold

**Fig. 2:**
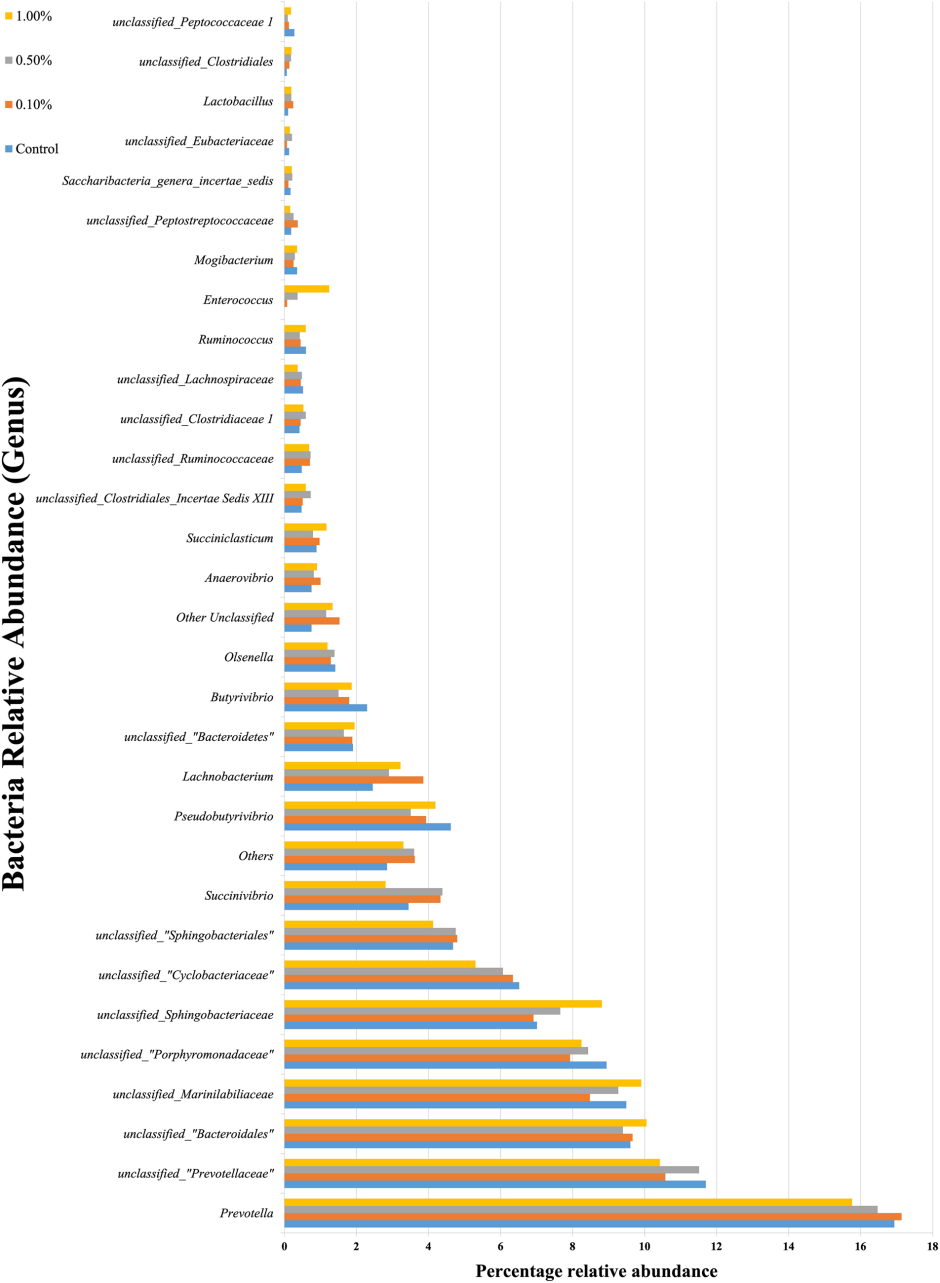
Bacterial genus-level compositions of the control and *Enterococcus faecium* SROD-supplemented rumen fermenta. The data portray genus-level 16S rRNA pyrotagged gene sequences. Sequences were classified using the RDP naive Bayesian rRNA Classifier with an 80% confidence threshold. The minor group in the panel is composed of genera with a percentage of reads < 0.4% of the total reads in all samples

**Fig. 3:**
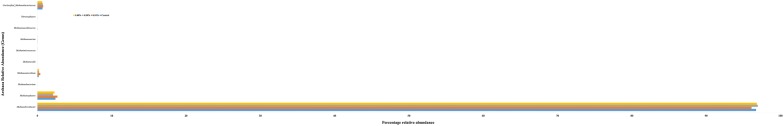
Archaeal genus-level compositions of the control and *Enterococcus faecium* SROD-supplemented rumen fermenta. The data portray genus-level 16S rRNA pyrotagged gene sequences. Sequences were classified using the RDP naive Bayesian rRNA Classifier with an 80% confidence threshold. The minor group in the panel is composed of genera showing a percentage of reads < 0.4% of the total reads in all samples

Bioinformatics analysis revealed that the bacterial sequences were predominantly affiliated with two phyla, *Bacteroidetes* and *Firmicutes* (Fig. 1), while the archaeal sequences were predominantly affiliated with phylum *Euryarchaeota*. Notably, a very low abundance of *Thaumarchaeota* was observed only in the group supplemented with 0.1% *E. faecium* SROD. At the genus level, *Prevotella* (15–17%) and *Methanobrevibacter* (96%) dominated the bacterial and archaeal communities’ composition of the in vitro rumen fermenta, respectively (Figs. 2 and 3). The relative abundance of *Anaerovibrio*, *Enterococcus*, *Lachnobacterium*, unclassified *Clostridiales Incertae Sedis XII*, unclassified *Clostridiaceae 1*, unclassified *Clostridiales*, and unclassified *Ruminococcaceae* also increased with supplementation of *E. faecium* SROD. Notably, the *Enterococcus* relative abundance increased with increasing inclusion rate of *E. faecium* SROD from 0% for the control, to 0.075%, 0.366%, and 1.240%, respectively, in each treatment group (Fig. 2). In addition, supplementation of 0.1% *E. faecium* SROD increased the relative abundance of *Methanomicrobium* to the greatest extent (0.386%), followed by 0.5% *E. faecium* SROD (0.211%); similar relative abundances were detected with 1.0% *E. faecium* SROD and the control of 0.184% and 0.175%, respectively.

## Discussion

### Effects of *E. faecium* SROD supplementation on rumen fermentation in vitro

The ruminal microbiome plays an important role not only in animal health and productivity but also in food and environmental safety. Enhancing the rumen microflora through probiotic supplementation stimulates fermentation. With prolonged culturing, our newly isolated probiotic strain *E. faecium* SROD displays full activity after 12 h of incubation (Kim et al. 2016); hence, in the present study, we collected and analyzed the fermentation parameters after 12 h of culture. The total gas production level is an indication of the fermentation rate. Since supplementation of 0.1% *E. faecium* SROD increased the gas production compared to the control, this probiotic appears to affect the fermentation rate of the rumen in vitro. This result was supported by Shi et al. (2017) claims that fermentation by inoculating *E. faecium* is an effective approach in improving the quality of corn-soybean meal mixed feed. The lower tendency of the pH with supplementation of *E. faecium* SROD could be related to the production of organic acids by the bacterium. Indeed, a previous study on *E. faecium* demonstrated that it increased the levels of organic acids such as acetate, propionate, and succinate (Ribeiro et al. 2009).

The lack of an effect on the ammonia–N concentration with supplementation of *E. faecium* SROD at all inclusion rates indicates that the probiotic does not influence the ruminal N-metabolism level (Pang et al. 2014). However, the methane concentration was significantly reduced with the lowest inclusion rate of 0.1% *E. faecium* SROD, which comparable to 0.5% *E. faecium* SROD inclusion rate. Moreover, we found that the group with the highest inclusion rate of 1.0% *E. faecium* SROD had a comparable methane concentration with that of the control, which is in opposition to our previous result of Kim et al. (2016) wherein inoculation of 1.0% *E. faecium* SROD significantly reduced the methane concentration. The difference in these results might be due to the different substrate used, which was maize silage in the previous study but was feed concentrate and rice straw at a 60:40 ratio in the present study. *E. faecium* SROD is a fumarate reductase-producing bacteria (Kim et al. 2016), which competes with methanogens in utilizing H_2_ in the rumen. H_2_ serves as electron donor for reduction of fumarate to succinate. Hence, lower methane concentration was observed in 0.1% *E. faecium* SROD inclusion.

Supplementation of *E. faecium* SROD increased the concentration of VFAs, and increasing inclusion rates of *E. faecium* SROD increased the acetate concentration. *E. faecium* SROD converts fumarate to succinate and thus, increase in available succinate for propionate production. Also, fumarate can be converted into propionate and acetate via different pathways (Demeyer and Henderickz 1967). Acetate is a precursor of milk constituents (Kleiber et al. 1952). Nocek and Kautz (2006) demonstrated an increase in milk yield by 2.3 L per cow per day following dietary supplementation with 5 × 10^9^ CFU of *E. faecium* and 2 × 10^9^ yeast (*Saccharomyces cerevisiae*) cells per cow per day. Moreover, the increased acetate concentration observed in the *E. faecium* SROD-supplemented treatment groups compared to the control was comparable that observed in our previous study on *E. faecium* SROD (Kim et al. 2016). However, a higher acetate concentration was observed in the present study. These results indicate that supplementation of *E. faecium* SROD as a probiotic can improve the milk fat and milk yield in dairy animals.

Higher concentrations of propionate, butyrate, and total VFAs concentrations were observed in the *E. faecium* SROD-supplemented groups than the control, which is line with the results reported by Kim et al. (2016) and Pang et al. (2014). It has been reported by Ribeiro et al. that *E. faecium* increased the levels of organic acids such as acetate, propionate, and succinate that made *E. faecium* as dietary supplement for domestic animals worldwide. Oetzel et al. (2007) reported that *E. faecium* plus *S. cerevisiae* increased milk fat percentages when used as direct fed microbe (DFM) for first lactation cows and increased milk protein percentages for second and greater lactation cows. Moreover, *E. faecium* with yeast as DFM increased dry matter intake, milk yield, and milk protein content during the postpartum period (Nocek et al. 2003).

Volatile fatty acids are important contributors to the overall performance of the animal because they improve growth, production, and health simultaneously. With these increase in VFAs means also increase in energy available for the animal, which explains the improvement of breast and legs yield, as well as the water holding capacity of meat but low abdominal fat deposition in dietary supplementation of *E. faecium* (Zampiga et al. 2018). The significant increase in butyrate concentration (Table 3) during fermentation is well known for many regulatory and immunological functions in cattle. Also, decreased in acetate to propionate ratio in this study indicates increased in the positive energy balance. Apás et al. (2010) reported that inclusion of a probiotic containing a mixture of *E. faecium* DDE 39, *Lactobacillus reuteri* DDL 19, *L. alimentarius* DDL 48, and *Bifidobacterium bifidum* DDBA resulted in improvement in average body weight by 9% when fed to goats for 8 weeks, commencing at 75 days of age, and increased body weight gain and improved feed use efficiency were observed with supplementation of *E. faecium*, *L. acidophilus*, *L. plantarum*, *L. salivarius*, *L. casei/paracasei*, or *Bifidobacterium* spp. to young calves compared with control groups (Frizzo et al. 2011).

### Effects of *E. faecium* SROD supplementation on rumen in vitro microbial abundance and community composition

Microorganisms inhabiting in the rumen contribute directly or indirectly to dietary organic matter degradation (Wang et al. 2017). *F. succinogenes* and *R. flavefaciens* are two of the major cellulolytic bacterial species found in the rumen, and *F. succinogenes* was reported to be present in greater quantities than *R. flavefaciens* (Koike and Kobayashi 2001), which was confirmed in the present study. Moreover, the lower quantities of methanogens observed with supplementation of *E. faecium* SROD, along with the increase of cellulolytic bacteria, *F. succinogenes* and *R. flavefaciens*, and general fungi quantities with supplementation of 0.1% *E. faecium* SROD support that the reduction in methane concentration was directly due to the activity of *E. faecium* SROD in reducing the abundance of methanogenic bacteria. Lower levels of supplementation of *E. faecium* SROD enhanced the cellulolytic bacteria *F. succinogenes* and *R. flavefaciens*, and the general fungi, which could explain the significant decrease in methane concentration. This decrease in methane production is similar in Chaucheyras-Durand et al. (2010) study when *F. succinogenes* was inoculated in lambs. *F. succinogenes* is a non-H_2_-producing species (Chaucheyras-Durand et al. 2010), which is a substrate for methane production and hence, lower methane concentration was observed in this study. However, increased supplementation of *E. faecium* SROD (1%) had comparable methane concentration to control but it tended decrease. This might be due to increase in population of *R. flavefaciens*, which might increase available H_2_ and electrons for methanogenesis.

Analysis of the rumen microbiome is of great importance for understanding the microbial ecosystem at large, which could be accomplished through determination of the microbial communities and their symbiosis. To best correlate and describe the results of in vitro rumen fermentation parameters with microbial abundance and community composition at greater resolution, we utilized a new and high-throughput molecular technique. Further adoption of high-throughput techniques can lay the foundation for new advancements in ruminant production by gaining a deeper-level microbial understanding of proven nutritional strategies (McCann et al. 2014). We conducted a sample-based rarefaction test to assess whether the samples and sequences provided efficient OTU coverage. The OTU is an operational definition of a species or a group of species that is often used when only DNA sequencing data are available. The alpha rarefaction curve constructed in this study became flattered to the right of the axis, which indicates that an efficient and reasonable number of reads had been used in the analysis; thus, additional sequencing was not necessary.

Through calculation of the number of OTUs and the measure of species richness estimators, we estimated the diversity within samples. The Chao index estimates the richness of the diversity, while the Shannon–Weaver index takes into account the number and evenness of species present. On the other hand, the Simpson index depicts probability of the that two randomly selected individuals in the habitat will belong to the same species. In this study, the pyrosequencing data demonstrated comparable bacterial or archaeal communities, in terms of diversity, richness, number, and evenness of species, among treatments. However, higher communities, diversity, richness, number and evenness of species were observed in bacteria than in archaea.

*Bacteroidetes* and *Firmicutes* were present at the highest relative abundance at the phylum level for all groups, which is consistent with the findings of Jami et al. (2013) and Wang et al. (2016). Naas et al. (2014) reported that *Bacteroidetes* specialize in lignocellulose degradation and are associated with butyrate production; however, *Firmicutes* represent the major butyrate-producing group of microbes (Naas et al. 2014). The dominance of *Euryarchaeota* in this study is in line with the results of Wang et al. (2016). *Thaumarchaeota*, which was observed only under supplementation with 0.1% *E. faecium* SROD, represents a group of chemolithoautotrophic ammonia-oxidizers (Spang et al. 2010) and is likely a dominant producer of the critical vitamin B12 (Doxey et al. 2015). Supplementation of *E. faecium* SROD also enhanced the growth of *Anaerovibrio*, *Enterococcus*, *Lachnobacterium*, unclassified *Clostridiales Incertae Sedis XII*, unclassified *Clostridiaceae 1*, unclassified *Clostridiales*, and unclassified *Ruminococcaceae* by increasing their relative abundances. The increased relative abundance of *Enterococcus* with increasing inclusion rates of *E. faecium* SROD indicates that *E. faecium* SROD grew well anaerobically and in symbiosis with other microbes.

The inclusion of 0.1% *E. faecium* SROD increased the concentrations of propionate and total VFAs but decreased the methane concentration during in vitro rumen fermentation. Also, a significant increase in butyrate concentration indicates regulatory and immunological functions in cattle. These findings were validated by the determination of the quantities of specific microbes related to the production of these components. In particular, the quantities of general fungi, *F. succinogenes*, and *R. flavefaciens* increased with the inclusion of 0.1% *E. faecium* SROD, while lower quantities of methanogens were observed in the treatment groups compared to the control. Using a pyrosequencing technique, we further demonstrated that supplementation of *E. faecium* SROD enhances the growth of *Anaerovibrio*, *Enterococcus*, *Lachnobacterium*, unclassified *Clostridiales Incertae Sedis XII*, unclassified *Clostridiaceae 1*, unclassified *Clostridiales*, and unclassified *Ruminococcaceae* by increasing their relative abundances.

Overall, these results demonstrate that *E. faecium* SROD is a potentially valuable feed additive for ruminal methane mitigation and to enhance the productivity of the ruminant. This will further help to reduce the use of harmful chemicals and antibiotics. To further evaluate the potential of *E. faecium* SROD, in vivo trial will be done to determine the growth performance, efficiency, and their effect on rumen microbiome and population of the animals as well as the methane production using Greenfeed technology.

## Data Availability

Not applicable.

## Abbreviations

NH_3_–N: ammonia–nitrogen
CFU: colony-forming units
GC: gas chromatography
OUT: operational taxonomic units
qRT-PCR: quantitative real-time polymerase chain reaction
SEM: standard error of the mean
VFAs: total volatile fatty acids
Con: Control
T1: 0.1% *E. faecium*
T2: 0.5% *E. faecium*
T3: 1.0% *E. faecium*
